# Right Ventricular and Chest Wall Perforation with Implantable Cardioverter-Defibrillator Lead with Lodgment into the Cutaneous Tissue of the Chest Wall

**DOI:** 10.19102/icrm.2017.080904

**Published:** 2017-09-15

**Authors:** Philip S. Carson, Jalag Garg, Talha Nazir, Babak Bozorgnia

**Affiliations:** ^1^Lehigh Valley Health Network, Allentown, PA

**Keywords:** Implantable cardioverter-defibrillator, lead perforation

## Abstract

Implantable cardioverter-defibrillator (ICD) lead perforation is a rare but serious complication of cardiac device implantation. Subacute (24 h to one month) and delayed (>1 month) presentations of rupture are also rare. Here we report a case of right ventricular perforation by a ventricular ICD lead in a 61-year-old man that was detected four months’ postimplantation. The lead was present out from his chest wall and was palpable beneath the skin.

## Introduction

Implantable cardioverter-defibrillator (ICD) lead perforation is a rare but serious complication of cardiac device implantation with a mean incidence of 0.82%.^1^ Perforation is usually accompanied by device malfunction, pericardial effusion, and possibly tamponade within the first few hours after implant. However, subacute (24 h to 1 month) and delayed (> 1 month) presentations also occur. Given its thin wall relative to the rest of the right ventricle, the apex is typically the perforated location.^2^ Diagnosis is made via transthoracic echocardiogram and chest computed tomography (CT).^3^ Lead extraction is performed by either transvenous manual traction or a hybrid surgical approach, both of which are safe and effective.^4^ Pericar-diocentesis or a pericardial window is necessary for cardiac tamponade.^4^ Here, we report a case of right ventricular perforation by a ventricular ICD lead in a 61-year-old man that was detected four months after implantation.

## Case presentation

**Figure 1:**
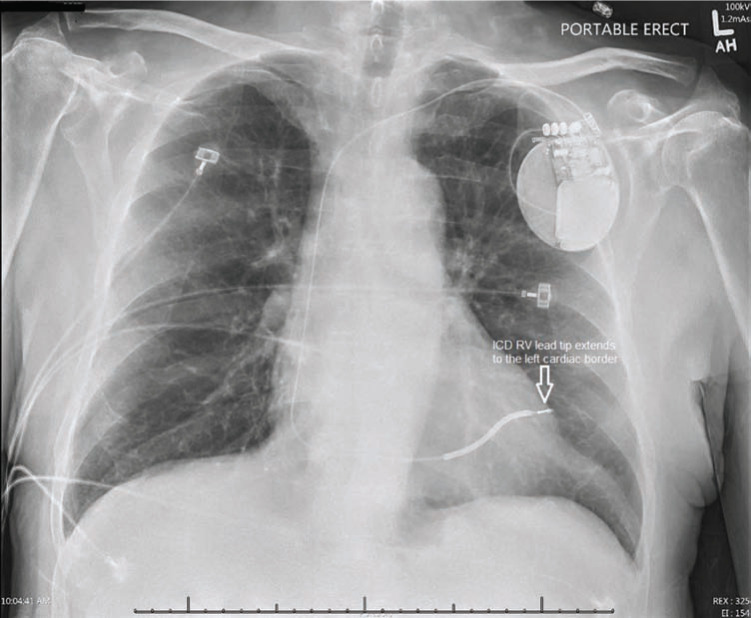
Chest X-ray indicating ICD lead misplacement.

**Figure 2:**
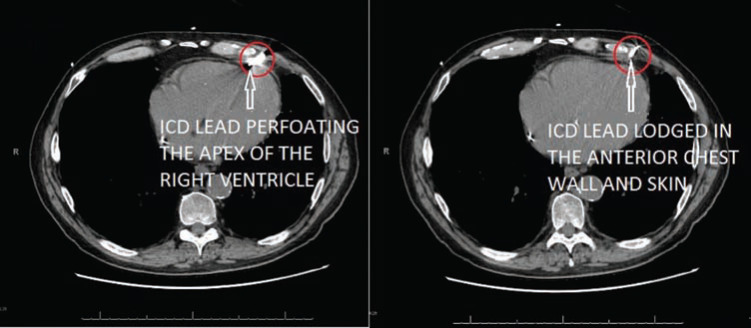
Chest CT showing the location of the lead tip without pericardial effusion, fluid collection, pneumomediastinum, or pneumothorax.

A 61-year-old man with a past medical history of non-ischemic cardiomyopathy, a left ventricular ejection fraction of 35% to 40%, permanent atrial fibrillation while on anticoagulation, and a single-chamber ICD implanted four months earlier in a different state as a secondary prevention device presented to an outside hospital with syncope. A chest X-ray suggested misplacement of the ICD lead **(Figure 1)**. A CT chest scan was performed and indicated lead perforation with the tip extending through the right ventricular apex into the right anterior abdominal wall. The lead tip was observed in the subcutaneous tissues anterior to the costochondral junction and appeared to be less than 1 cm from the skin surface. There was no pericardial effusion or fluid collection surrounding the distal aspect of the lead and no evidence of pneumomediastinum or pneumothorax **(Figure 2)**.

The patient was transferred to our facility for further management. Upon arrival, he was in no distress, with a heart rate of 86 bpm and a blood pressure rate of 132/ 100 mmHg. Physical examination was significant for a firm, pointed object along the fifth intercostal space at the midclavicular line. A transthoracic echocardiogram revealed the ICD lead in the right ventricle, with trace pericardial effusion.

Interrogation of the present Inogen™ EL ICD (Boston Scientific, Natick, MA, USA) showed an impedance of 419 ohm and a pacing threshold of 20V@ 10 ms, as well as numerous device-labeled episodes of ventricular tachycardia and fibrillation, which were determined to be noise. The records from the facility where the device was implanted were not available for comparison. The patient was subsequently scheduled for device extraction in the operating room.

**Figure 3:**
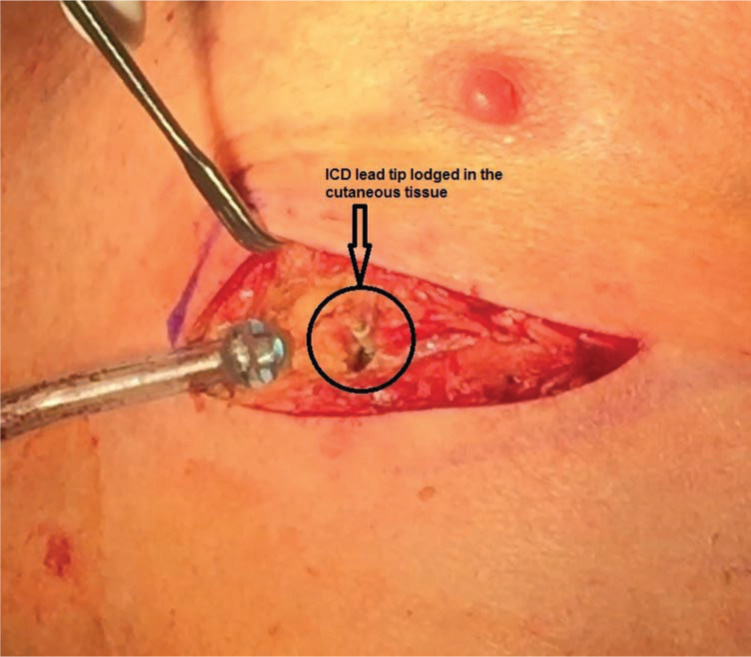
Surgical exposure of the ICD lead tip in the cutaneous tissue beneath the left nipple. It was observed that the lead was beating with the heart.

During extraction, the device generator was disconnected from the leads and explanted, the leads were dissected down to the tie-down sleeve, and fluoroscopy was performed to locate the protruding ICD lead on the chest wall. The lead location was then marked on the skin. Notably, the lead itself was actually moving in and out of the chest wall with each heartbeat **(Figure 3)**. Following skin marking, the lead tip was cut, and the lead was manually pulled from the pocket under fluoroscopy. An intraoperative transesophageal echo-cardiogram demonstrated no pericardial effusion. A new ICD lead (INOGEN™ EL; Boston Scientific, Natick, MA, USA) was then implanted into the apical right ventricular septum. The patient had an uneventful postoperative course and was discharged two days after surgery.
